# Supplementary figures and images for: Diagnostic accuracy of a novel tuberculosis point-of-care urine lipoarabinomannan assay for people living with HIV: A meta-analysis of individual in- and outpatient data
Source: PLoS Med. 2020 May 1;17(5):e1003113. doi: 10.1371/journal.pmed.1003113 (PMC7194366; doi:10.1371/journal.pmed.1003113)

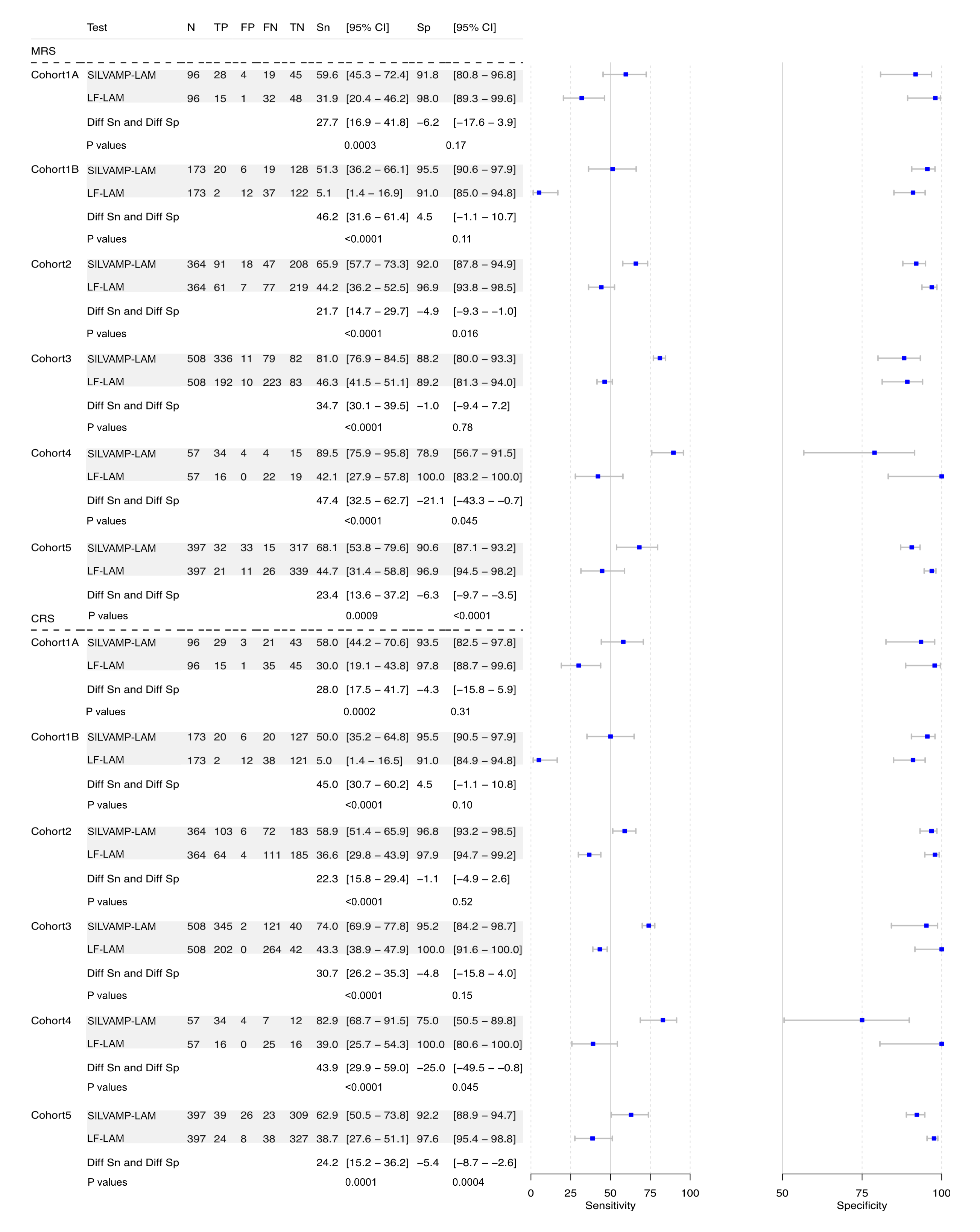

Supplement: S1 Fig — (TIFF) [file pmed.1003113.s002.tiff]

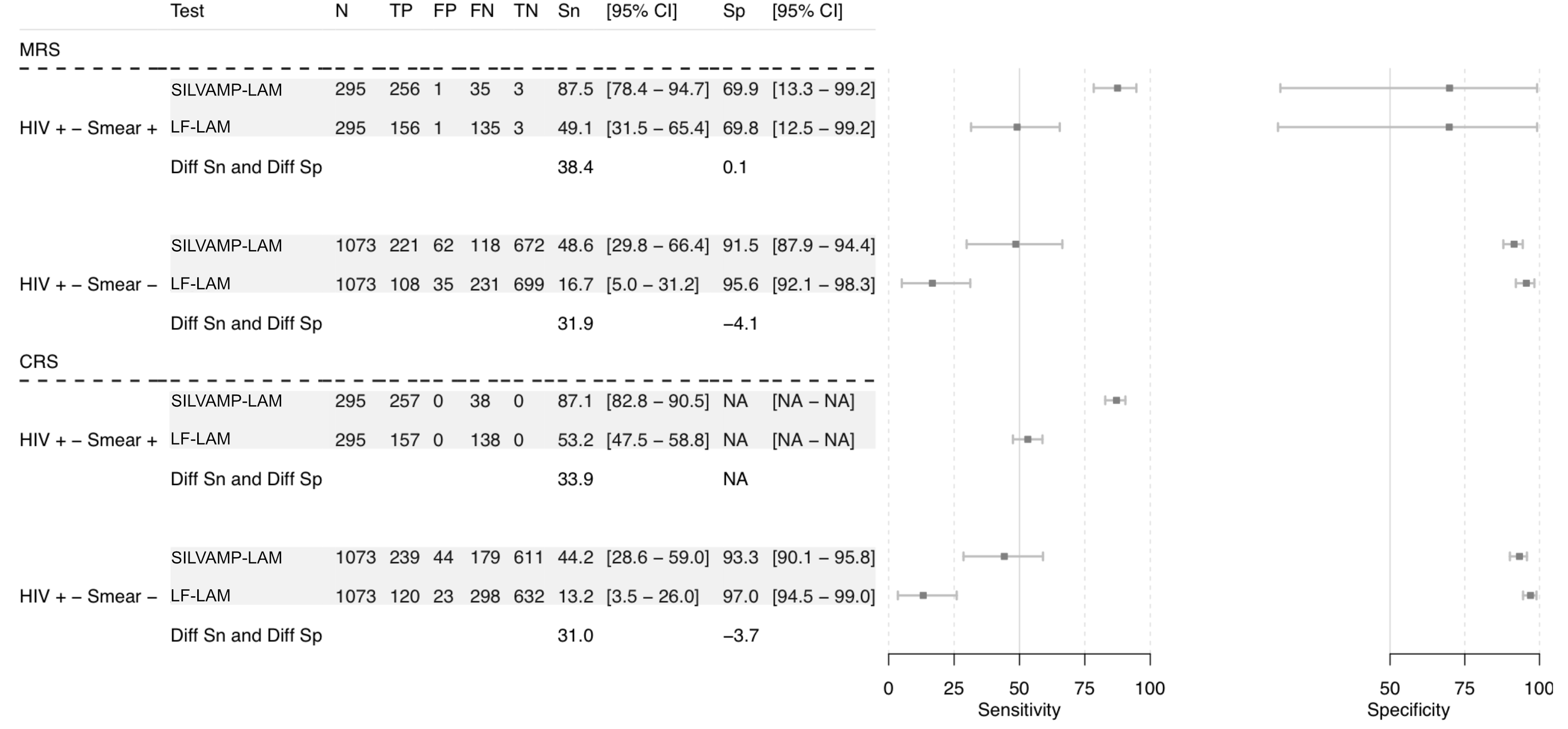

Supplement: S2 Fig — (TIFF) [file pmed.1003113.s003.tiff]
