# Supplementary material for: Diagnostic accuracy of a novel tuberculosis point-of-care urine lipoarabinomannan assay for people living with HIV: A meta-analysis of individual in- and outpatient data
Source: PLoS Med. 2020 May 1;17(5):e1003113. doi: 10.1371/journal.pmed.1003113 (PMC7194366; doi:10.1371/journal.pmed.1003113)
Supplement: S1 Table — (DOCX) [file pmed.1003113.s006.docx]

# S1 Table. Study population, setting and location, eligibility, and inclusion and exclusion criteria used for the studies

| **Cohort name** | **Cohort1 (A and B)** | **Cohort2** | **Cohort3** | **Cohort4** | **Cohort 5** |
| --- | --- | --- | --- | --- | --- |
| **Study population** | Adults suspected of having active TB disease | Unselected adults regardless of presenting symptoms | Adults suspected of having active TB disease | Adults suspected of having active TB disease | Unselected adults with HIV regardless of presenting symptoms |
|  | High HIV prevalence setting | High HIV prevalence setting | High HIV prevalence setting | High HIV prevalence setting | High TB/HIV burden setting |
| **Setting & location** | South Africa, Cape Town   - Khayelitsha Hospital - Town Two Clinic - Nolungile Clinic - Inpatient admissions and outpatients | South Africa, Cape Town   - GF Jooste Hospital - Public sector district hospital that served township communities - Inpatient admissions | South Africa, Cape Town   - Khayelitsha Hospital - Public sector district hospital that serves township communities - Inpatient admissions | Vietnam, Ho Chi Minh   - Pham Ngoc Thach Hospital - Public sector district hospital - Outpatient | Ghana, Accra   - Korle-Bu Teaching Hospital, Fevers Unit - Public referral hospital in the capital city of Ghana - Inpatients and Outpatients |
| **Eligibility criteria** | Adults (≥18 years) presenting with symptomatic pulmonary disease thought to have TB | Unselected HIV-infected adults (≥18 years) regardless of clinical presentation admitted to adult medical wards at Jooste Hospital | HIV-infected adults (≥18 years) with CD4 counts ≤350 cells/µL and suspected to have active TB | Adults (≥18 years) presenting with symptomatic pulmonary disease thought to have TB | Unselected HIV-infected adults (≥18 years) presenting with or without TB related signs and symptoms assessed prior to initiation of ART |
| **Inclusion criteria** | - Informed consent from patient - Suspected to have active TB based on clinical presentation - Production of adequate quantity of sputum | - Informed consent from patient - Regardless of presenting symptoms or reason for hospital admission - HIV seropositive | - Informed consent as per study protocol - Suspected to have active TB based on clinical presentation - HIV seropositive - CD4≤ 350 cells/µL | - Informed consent from patient - Suspected to have active TB based on clinical presentation - Production of adequate quantity of sputum | - Informed consent from patient - HIV-positive - Referred for ART initiation - Regardless of presenting with or signs and symptoms and reason for initiation of ART |
| **Exclusion criteria** | - Participants receiving any anti-tuberculosis medication in the 60 days prior to enrolment - Participants with only extra-pulmonary disease were excluded | Patients that already received treatment for an existing diagnosis of TB at the time of admission | - Three or more doses of TB treatment received during the admission or has been on TB treatment within 1 month of admission - Pregnant | - Participants receiving any anti-tuberculosis medication in the 60 days prior to enrolment - Participants with only extra-pulmonary disease were excluded | Patients that had received more than 2 days of TB treatment within the last 3 months |

| **Cohort name** | **Cohort1 (A and B)** | **Cohort2** | **Cohort3** | **Cohort4** | **Cohort 5** |
| --- | --- | --- | --- | --- | --- |
| **Enrolment period** | Feb 2017 – Aug 2017 | June 2012 – Oct 2013 | Jan 2014 – Oct 2016 | Sep 2016 – July 2017 | Jan 2013 and March 2014 |
| **Participants considered for retrospective urinary LAM testing** | The full cohort (n=528) of the prospective cohort study. Patients with no urine available (n=14) or withdrawn (n=6) were excluded from the analysis. | The full cohort (n=420) of the prospective, consecutive cohort study. | The full cohort (n=659) of the prospective, observational cohort study. | The full cohort (n=239). | The full outpatient cohort (n=498) of the prospective, consecutive cohort study. Patients with negative HIV status (n=2), on TB treatment (n=4) and re-admission (n=1) were excluded. 70 inpatient participants were excluded from analyses as data became available later. |
| **Study protocol, Principle Investigator (PI) and References** | Protocol: FIND Reference Materials Collection Protocol Version 14.0.  PI: Prof. Dr. Mark Nicol  Protocol available on request. | Protocol: “Systematic Screening for HIV associated Tuberculosis in Patients Admitted to GF Jooste District Hospital, Cape Town”.  PI: Prof. Dr. Stephen D. Lawn Protocol available on request. | Protocol: “Defining Interventions to reduce mortality in severe HIV-associated TB” Version 4.0.  PI: Prof. Dr. Graeme Meintjes  Protocol available in Publication | Protocol: FIND Reference Materials Collection Protocol date 06 May 2015.  PI: Dr. Ha Dang Thi Minh  Protocol available on request. | Protocol: “The HIV-associated Tuberculosis Epidemic in Ghana: Potential for Improved Tuberculosis Case detection”.  PI: MD, PHD Stephanie Bjerrum  Protocol available on request. |
