# Supplementary material for: Diagnostic accuracy of a novel tuberculosis point-of-care urine lipoarabinomannan assay for people living with HIV: A meta-analysis of individual in- and outpatient data
Source: PLoS Med. 2020 May 1;17(5):e1003113. doi: 10.1371/journal.pmed.1003113 (PMC7194366; doi:10.1371/journal.pmed.1003113)
Supplement: S2 Table — (DOCX) [file pmed.1003113.s007.docx]

# S2 Table. Specimen collection and testing flow by cohort

|  | | | | |
| --- | --- | --- | --- | --- |
| **Cohort 1** | | | | |
| **Time point** | **Clinical** | **Urine** | **Blood** | **Sputum** |
| **Enrollment Day1** | - Informed consent - Inclusion and exclusion - Clinical questionnaire | **Pooled urine**  **(Urine1 and Urine2)**   - Sample collection for retrospective testing (SILVAMP-LAM & LF-LAM) - Urinary Xpert (30-40ml) - Urinary dipstick - LF-LAM (fresh) | **Venous blood**   - Blood culture (BACTEC Myco F/Lytic) - HIV testing (RDT) - CD4 cell count (flow cytometry) | **Sputum1 and Sputum2**   - Smear microscopy - Xpert - Solid culture (LJ) / Speciation - Liquid culture (MGIT) / Speciation MGIT - DST |
| **Enrollment Day2** |  | **Urine 3**   - Urinary dipstick - Alere LAM (fresh) |  | **Sputum 3**   - Smear microscopy - Xpert - Solid culture (LJ) / Speciation - Liquid culture (MGIT) / Speciation MGIT - DST |
| **Follow-up (8 weeks)** | - Clinical questionnaire |  |  | **Sputum 4**   - Smear microscopy - Xpert - Solid culture (LJ) / Speciation - Liquid culture (MGIT) / Speciation MGIT - DST |

| **Cohort 2** | | | | | |
| --- | --- | --- | --- | --- | --- |
| **Time point** | **Clinical** | **Urine** | **Blood** | **Sputum** | **Other specimen** |
| **Enrollment Day1** | - Informed consent - Inclusion and exclusion - Clinical questionnaire | **Urine1**   - Sample collection for retrospective testing (SILVAMP-LAM & LF-LAM) - Urinary Xpert (2 ml) - Urinary Xpert (30-40 ml) | **Venous Blood**   - Blood culture (BACTEC Myco F/Lytic) - HIV testing (RDT) - CD4 cell count (flow cytometry) | **Sputum 1 and Sputum 2 (sputum induction if necessary)**   - Smear microscopy - Xpert - Liquid culture / Speciation - Liquid culture (MGIT) / Speciation MGIT - DST |  |
| **Throughout admission period, if available (including samples from routine)** | Clinical information |  | **Venous Blood**   - Blood culture (BACTEC Myco F/Lytic) | **Sputum(s)**   - Xpert - Culture | **Other non-respiratory samples** for Xpert or culture including (ascitic fluid, bone marrow, cerebrospinal fluid, fine needle aspirate, gastric lavage, pus, pleural fluid, stool, urine) |
| **Follow-up (12 weeks)** | Clinical information |  |  |  |  |

| **Cohort 3** | | | | | |
| --- | --- | --- | --- | --- | --- |
| **Time point** | **Clinical** | **Urine** | **Blood** | **Sputum** | **Other specimen** |
| **Before the 3^rd^ dose of TB treatment** | - Informed consent (as per protocol) - Inclusion and exclusion - Clinical questionnaire - Clinical examination | - Sample collection for retrospective testing (SILVAMP-LAM and LF-LAM) - Urinary Xpert (30-40 ml) | **Venous Blood**   - Blood culture (BACTEC Myco F/Lytic) - GenoType MTBDR*plus* assay (Hain Lifesciences) on positive cultures - HIV viral load - CD4 cell count | **Sputum induction performed if necessary**   - Smear Microscopy - Xpert - Liquid Culture (MGIT) - GenoType MTBDR*plus* assay (Hain Lifesciences) on positive cultures |  |
| **Throughout admission period, if available (including samples from routine)** | Clinical information | **Urine**  Culture | **Venous Blood**  Blood culture (BACTEC Myco F/Lytic) | **Sputum(s)**   - Xpert - Culture | **Other non-respiratory samples** for Xpert or culture including ascitic fluid, cerebrospinal fluid, fine needle aspirate of nodes, puss, pleural fluid and pericardial fluid |
| **Follow-up (12 weeks)** | Clinical information |  |  |  |  |

|  | | | | |
| --- | --- | --- | --- | --- |
| **Cohort 4** | | | | |
| **Time point** | **Clinical** | **Urine** | **Blood** | **Sputum** |
| **Enrollment Day1** | - Informed consent - Inclusion and exclusion - Clinical questionnaire | **Urine 1** Sample collection for retrospective testing (SILVAMP-LAM and LF-LAM) | **Venous blood**   - HIV testing (RDT) - CD4 cell count (flow cytometry) | **Sputum 1**   - Smear microscopy - Xpert - Solid culture (LJ) / Speciation - Liquid culture (MGIT) / Speciation MGIT - DST |
| **Enrollment Day2** |  |  |  | **Sputum 2**   - Smear microscopy - Solid culture (LJ) / Speciation - Liquid culture (MGIT) / Speciation MGIT - DST |
| **Follow-up (8 weeks)** | Clinical questionnaire |  |  | **Sputum 3**   - Smear microscopy - Solid culture (LJ) / Speciation - Liquid culture (MGIT) / Speciation MGIT - DST |

|  | | | |
| --- | --- | --- | --- |
| **Cohort 5** | | | |
| **Time point** | **Clinical** | **Urine** | **Sputum** |
| **Enrollment Day1** | - Informed consent - Inclusion and exclusion - Clinical standardized questionnaire - Clinical basic investigation | - LF-LAM (Fresh) - Urine dipstick - Xpert MTB/RIF (6mL, on stored samples) | **Sputum 1**   - Smear microscopy (ZN and FM) - Xpert MTB/RIF - Solid culture (LJ) - Liquid culture (MGIT) - Identification of mycobacterial cultures by microscopy for AFB and MTBC confirmed by Capilia immunoassay - Mycobacterial speciation by GenoType MTBC/CS/AS on positive mycobacterial cultures - DST for first line TB drugs |
| **Enrollment Day2** |  | - LF-LAM (Fresh) - Urine dipstick - Xpert MTB/RIF (6- mL, on stored samples, if not done on Day1 specimen) | **Sputum 2 (early morning)**   - Smear microscopy (ZN and FM) - Xpert MTB/RIF - Solid culture (LJ) - Liquid culture (MGIT) - Identification of mycobacterial cultures by microscopy for AFB and MTBC confirmed by Capilia immunoassay - Mycobacterial speciation by GenoType MTBC/CS/AS on positive mycobacterial cultures - DST (MTBC) for first line TB drugs |
| **Follow-up (6 months)** | - Record review of clinical consultations - If no records, personal communication for vital status and treatment start (ART; TB treatment) |  |  |
