# Supplementary material for: Diagnostic accuracy of a novel tuberculosis point-of-care urine lipoarabinomannan assay for people living with HIV: A meta-analysis of individual in- and outpatient data
Source: PLoS Med. 2020 May 1;17(5):e1003113. doi: 10.1371/journal.pmed.1003113 (PMC7194366; doi:10.1371/journal.pmed.1003113)
Supplement: S3 Table — (DOCX) [file pmed.1003113.s008.docx]

# S3 Table. Specimen storage

| **Cohort** | **Place of urine collection** | **Place of LF-LAM and SILVAMP-LAM testing** | **Time of collection** | **Time of testing** | **Storage temperature** | **Shipment temperature** |
| --- | --- | --- | --- | --- | --- | --- |
| Cohort1A | South Africa | South Africa | Feb 2017 to Aug 2017 | Apr 2018 to May 2018 | −80°C | Not shipped |
| Cohort1B | South Africa | Japan | Feb 2017 to Aug 2017 | Jan 2019 to Mar 2019 | −80°C | Dry ice (-78°C) |
| Cohort2 | South Africa | South Africa | Jun 2012 to Oct 2013 | Apr 2018 to May 2018 | −20°C | Not shipped |
| Cohort3 | South Africa | South Africa | Jan 2014 to Oct 2016 | Apr 2018 to May 2018 | −80°C | Not shipped |
| Cohort4 | Vietnam | Japan | Sep 2016 to Jul 2017 | Jan 2019 to Mar 2019 | −80°C | Dry ice (-78°C) |
| Cohort5 | Ghana | Japan | Jan 2013 to Mar 2014 | Jan 2019 to Mar 2019 | −20°C | Dry ice (-78°C) |
