# Supplementary material for: Diagnostic accuracy of a novel tuberculosis point-of-care urine lipoarabinomannan assay for people living with HIV: A meta-analysis of individual in- and outpatient data
Source: PLoS Med. 2020 May 1;17(5):e1003113. doi: 10.1371/journal.pmed.1003113 (PMC7194366; doi:10.1371/journal.pmed.1003113)
Supplement: S4 Table — (DOCX) [file pmed.1003113.s009.docx]

# S4 Table. Diagnostic categories by cohort

| **Category** | **Cohort1, 4** | **Cohort2** | **Cohort3** | **Cohort5** |
| --- | --- | --- | --- | --- |
| **Definite TB** | **Any culture or any Xpert (baseline) positive for MTB**  ≥1 Positive culture (solid, liquid, sputum or blood) & confirmed MTB complex at baseline (Cross-contamination: A single LJ culture with ≤ 20 colonies or a single MGIT culture with MTB growth ≥28 days per patient are excluded from analysis)  OR  ≥1 Positive Xpert (sputum or urine) at baseline | **Any culture or any Xpert positive for MTB from any anatomic sample**  ≥1 Positive culture (solid, liquid) & confirmed MTB complex from any clinical sample during enrolment admission  OR  ≥1 Positive Xpert (sputum, urine or any other clinical sample during enrolment admission | **Any culture or any Xpert positive for MTB**  ≥1 Positive culture (solid, liquid) & confirmed MTB complex from any clinical sample during enrolment admission  OR  ≥1 Positive Xpert (sputum or urine or any other clinical sample) during enrolment admission | **Any culture or any Xpert (baseline) positive for MTB**  ≥1 Positive culture (solid, liquid, sputum) & confirmed MTB complex at baseline  OR  ≥1 Positive Xpert (sputum or urine) at baseline |
| **Possible TB** | **Any patient not meeting definite TB or Not TB classification who is started on TB treatment or has positive laboratory findings on follow-up**  Empiric TB treatment started by the healthcare provider  OR  Positive sputum culture and/or sputum Xpert and/or sputum smear on follow-up | Any patient not meeting definite TB or Not TB classification,  who either has clinical/radiographic features suggestive of TB  OR  Any patient who is started on TB treatment | Any patient not meeting definite TB or Not TB classification who is started on TB treatment and TB treatment not stopped due to alternative diagnosis  AND  Alive at 12 weeks, symptoms improved on TB treatment | **Any patient not meeting definite TB or Not TB classification who is started on TB treatment or has positive laboratory findings on follow-up**  Empiric TB treatment started by the healthcare provider  OR  Positive sputum culture and/or sputum Xpert and/or sputum smear on follow-up |
| **Not TB** | **All microscopy, culture and Xpert tests negative for MTB, not started on TB treatment, recovers and has negative follow-up tests**  All culture negative (sputum, blood, incl. follow-up, with at least 2 LJ or MGIT with no culture growth after >56 days and >42 days)  AND  At least 2 valid negative culture or Xpert results from 2 or more independent samples obtained from 2 or more different anatomic sites, such as blood, sputum, or urine  AND  All Xpert negative (incl. follow-up)  AND  All smear microscopy results negative (incl. follow-up)  AND  Treatment not initiated by healthcare providers  AND  Improvement or full recovery at 8-week follow-up in the absence of TB treatment | All microscopy, cultures and Xpert tests negative for MTB, not started on TB treatment during hospitalization, and alive at 90 days  AND  At least 2 valid (and negative) culture or Xpert results available (at least 1 of which was a culture result) | All microscopy, cultures and Xpert tests negative for MTB, not started on TB treatment, alive at 12 weeks and not on TB treatment at 12 weeks.  AND  At least 1 valid (and negative) culture result available | **All microscopy, culture and Xpert tests negative for MTB, not started on TB treatment, recovers and has negative follow-up tests**  All cultures negative (sputum, blood, incl. follow-up where available)  AND  All Xpert negative (sputum, urine, incl. follow-up where available)  AND  All smear microscopy negative (sputum incl. follow-up where available)  AND  Treatment not initiated by healthcare providers  AND  Improvement or full recovery of symptoms at 2 months of follow-up in the absence of TB treatment |
| **Unclassifiable** | **All participants that do not fall into groups “Definite TB”, “Not TB” or “Possible TB”**  i.e.:  No symptom resolution on follow-up (same or worse) for baseline negative participants  OR  Loss to follow-up for baseline negative participants  OR  Passed away for baseline negative participants  OR  Insufficient laboratory results (i.e. participants with <2 valid cultures)  OR  Baseline smear microscopy positive but culture and Xpert negative | **All participants that do not fall into group “Definite TB”, “Not TB” or “Possible TB”**  i.e.:  Participants without definite or probable TB but died within 90 days  OR  Participants without definite or probable TB but were LTFU within 90 days  OR  Patient with baseline smear microscopy positive but culture and Xpert negative and not falling into the “possible TB” category (i.e., not started on TB treatment) | **All participants that do not fall into group “Definite TB”, “Not TB” or “Possible TB”**  i.e.:  Without definite TB but started TB treatment, while still on TB treatment, deteriorated/died/lost to follow-up or the symptoms not improved at 12 weeks.  OR  Loss to follow-up for baseline negative participants  OR  Died before start of TB treatment for baseline negative participants  OR  Insufficient laboratory results (i.e. participants with no valid cultures)  OR  Baseline smear microscopy positive but culture and Xpert negative and not falling into the “possible TB” category  OR  All microscopy, cultures and Xpert tests negative for MTB, started on TB treatment and TB treatment stopped during the same admission due to alternative diagnosis being made | **All participants that do not fall into groups “Definite TB”, “Not TB” or “Possible TB”**  i.e.:  No symptom resolution at follow-up (same or worse) in patients with a mycobacterial work-up that are negative at baseline or in follow-up at 2-3months  OR  Loss to follow-up at 2-3months (patients with a mycobacterial work-up that is negative at baseline)  OR  Passed away (patients with a mycobacterial work-up that is negative at baseline)  OR  Insufficient laboratory results  OR  Baseline smear microscopy positive but culture and Xpert negative |
