# Supplementary material for: Diagnostic accuracy of a novel tuberculosis point-of-care urine lipoarabinomannan assay for people living with HIV: A meta-analysis of individual in- and outpatient data
Source: PLoS Med. 2020 May 1;17(5):e1003113. doi: 10.1371/journal.pmed.1003113 (PMC7194366; doi:10.1371/journal.pmed.1003113)
Supplement: S5 Table — (DOCX) [file pmed.1003113.s010.docx]

#

# S5 Table. Definition and examples of “unclassifiable” category

| **Unclassifiable** |  | |  |
| --- | --- | --- | --- |
| BL-, no Rx, died |  | BL-, baseline TB negative  Rx, anti-tuberculosis treatment  FU, follow-up  LTFU, lost to follow-up  Smear+, sputum smear microscopy positive  Culture-, mycobacterial culture negative  Diag., diagnosis | |
| BL-, Rx, died |  |  |  |
| BL-, no Rx, LTFU |  |  |  |
| BL-, Rx, alternative diag., Rx stopped |  |  |  |
| BL-, Rx, deteriorated |  |  |  |
| BL-, Rx, symptoms not improved |  |  |  |
| BL-, no Rx, symptoms not improved |  |  |  |
| No valid culture/Xpert results |  |  | |
| BL-, Rx, LTFU |  |  | |
| BL-, no Rx initiated, but Rx initiated later |  |  | |
| Smear+, Culture- |  |  | |
