# Supplementary material for: Diagnostic accuracy of a novel tuberculosis point-of-care urine lipoarabinomannan assay for people living with HIV: A meta-analysis of individual in- and outpatient data
Source: PLoS Med. 2020 May 1;17(5):e1003113. doi: 10.1371/journal.pmed.1003113 (PMC7194366; doi:10.1371/journal.pmed.1003113)
Supplement: S6 Table — (DOCX) [file pmed.1003113.s011.docx]

# S6 Table. Two-by-two table of SILVAMP-LAM versus LF-LAM among “definite TB” patients

| **N=724** | **LF-LAM**  **positive** | **LF-LAM**  **negative** |
| --- | --- | --- |
| **SILVAMP-LAM positive** | 302 | 239 |
| **SILVAMP-LAM negative** | 5 | 178 |
