# Supplementary material for: Diagnostic accuracy of a novel tuberculosis point-of-care urine lipoarabinomannan assay for people living with HIV: A meta-analysis of individual in- and outpatient data
Source: PLoS Med. 2020 May 1;17(5):e1003113. doi: 10.1371/journal.pmed.1003113 (PMC7194366; doi:10.1371/journal.pmed.1003113)
Supplement: S8 Table — (DOCX) [file pmed.1003113.s013.docx]

# S8 Table. Analysis by cohort, smear status, and CD4 group for all HIV-positive inpatients

| **Cohort** | **Smear status** | **CD4 (cells/µL)** | | | | | **Total smear** |
| --- | --- | --- | --- | --- | --- | --- | --- |
|  |  | **≤ 100** | **101-200** | **201-350** | **≥ 350** | **UK** |  |
| All inpatients  (n=968) | Both | 516 (53%) | 216 (22%) | 130 (14%) | 101 (10%) | 5 (1%) |  |
|  | Smear - | 272 (53%) | 134 | 62 | 45 | 2 | 515 (53%) |
|  | Smear + | 158 (31%) | 39 | 20 | 7 | 2 | 226 (23%) |
|  | UK smear | 86 (16%) | 43 | 48 | 49 | 1 | 227 (24%) |
| 1A  (n=96) | Both | 44 (46%) | 19 (20%) | 11 (11%) | 19 (19%) | 3 (3%) |  |
|  | Smear - | 28 (64%) | 15 | 8 | 14 | 1 | 66 (69%) |
|  | Smear + | 16 (36%) | 4 | 3 | 5 | 2 | 30 (31%) |
|  | UK smear | 0 | 0 | 0 | 0 | 0 | 0 |
| 2  (n=364) | Both | 135 (37%) | 82 (23%) | 63 (17%) | 82 (23%) | 2 (1%) |  |
|  | Smear - | 35 | 30 | 15 | 31 | 1 | 112 (31%) |
|  | Smear + | 14 | 9 | 1 | 2 | 0 | 26 (7%) |
|  | UK smear | 86 | 43 | 47 | 49 | 1 | 226 (62%) |
| 3  (n=508) | both | 337 (66%) | 115 (23%) | 56 (11%) | 0 | 0 |  |
|  | Smear - | 209 | 89 | 39 | 0 | 0 | 337 (66%) |
|  | Smear + | 128 | 26 | 16 | 0 | 0 | 170 (33%) |
|  | UK smear | 0 | 0 | 1 | 0 | 0 | 1 (1%) |
