# Supplementary material for: Diagnostic accuracy of a novel tuberculosis point-of-care urine lipoarabinomannan assay for people living with HIV: A meta-analysis of individual in- and outpatient data
Source: PLoS Med. 2020 May 1;17(5):e1003113. doi: 10.1371/journal.pmed.1003113 (PMC7194366; doi:10.1371/journal.pmed.1003113)
Supplement: S9 Table — (DOCX) [file pmed.1003113.s014.docx]

#

# S9 Table. Analysis by cohort, smear status, and CD4 group for all HIV-positive outpatients

| Cohort | Smear status | **CD4 (cells/µL)** | | | | | Total (%) |
| --- | --- | --- | --- | --- | --- | --- | --- |
|  |  | **≤ 100** | **101-200** | **201-350** | **≥ 350** | **UK** |  |
| All outpatients  (n=627) | Both | 161 (25%) | 103 (17%) | 117 (19%) | 233 (37%) | 13 (2%) |  |
|  | Smear - | 128 | 93 | 105 | 221 | 11 | 558 (89%) |
|  | Smear + | 33 | 10 | 12 | 12 | 2 | 69 (11%) |
| 1B  (n=173) | Both | 15 (9%) | 13 (7%) | 33 (19%) | 112 (65%) | 0 |  |
|  | Smear - | 11 | 10 | 27 | 104 | 0 | 152 (88%) |
|  | Smear + | 4 | 3 | 6 | 8 | 0 | 21 (12%) |
| 5  (n=57) | Both | 31 (54%) | 8 (14%) | 3 (5%) | 12 (21%) | 3 (5%) |  |
|  | Smear - | 13 | 5 | 3 | 10 | 2 | 33 (58%) |
|  | Smear + | 18 | 3 | 0 | 2 | 1 | 24 (42%) |
| 6  (n=97) | Both | 115 (29%) | 82 (20%) | 81 (20%) | 109 (27%) | 10 (4%) |  |
|  | Smear - | 104 | 78 | 75 | 107 | 9 | 373 (94%) |
|  | Smear + | 11 | 4 | 6 | 2 | 1 | 24 (6%) |
