# Supplementary material for: Diagnostic accuracy of a novel tuberculosis point-of-care urine lipoarabinomannan assay for people living with HIV: A meta-analysis of individual in- and outpatient data
Source: PLoS Med. 2020 May 1;17(5):e1003113. doi: 10.1371/journal.pmed.1003113 (PMC7194366; doi:10.1371/journal.pmed.1003113)
Supplement: S10 Table — (DOCX) [file pmed.1003113.s015.docx]

# S10 Table. Sensitivity analysis of diagnostic accuracy for all PLHIV (including “unclassifiable” patients) by MRS and CRS

For the sensitivity analysis, those who were unclassifiable were included in the NON-TB category within the MRS and separately in the TB category within the CRS.

| **MRS** |  |  |  |  |  |  |  |  |  |  |
| --- | --- | --- | --- | --- | --- | --- | --- | --- | --- | --- |
| **(A) Original Analysis** | |  |  |  |  |  |  |  |  |  |
| **MRS positive (Definite TB) / MRS negative (Possible TB, Not TB)** | | | | |  |  |  |  |  |  |
|  | **Test** | **N** | **TP** | **FP** | **FN** | **TN** | **Sn %** | **95% CI** | **Sp %** | **95% CI** |
| **All HIV+ MRS** | SILVAMP-LAM | 1595 | 541 | 76 | 183 | 795 | 70.7 | [59 - 80.8] | 90.9 | [87.2 - 93.7] |
|  | LF-LAM | 1595 | 307 | 41 | 417 | 830 | 34.9 | [19.5 - 50.9] | 95.3 | [92.2 - 97.7] |
|  | Δ Sn and Δ Sp |  |  |  |  |  | 35.8 |  | -4.4 |  |
|  |  |  |  |  |  |  |  |  |  |  |
| **(B) Sensitivity Analysis** | |  |  |  |  |  |  |  |  |  |
| **MRS positive (Definite TB) / MRS negative (Possible TB, Not TB, Unclassifiable)** | | | | | |  |  |  |  |  |
|  | **Test** | **N** | **TP** | **FP** | **FN** | **TN** | **Sn %** | **95% CI** | **Sp %** | **95% CI** |
| **All HIV+ MRS** | SILVAMP-LAM | 1795 | 541 | 98 | 183 | 973 | 70.5 | [59.3 - 80.1] | 90.5 | [86.3 - 93.8] |
|  | LF-LAM | 1795 | 307 | 59 | 417 | 1012 | 34.9 | [19.6 - 51] | 94.5 | [91.3 - 97.2] |
|  | Δ Sn and Δ Sp |  |  |  |  |  | 35.6 |  | -4.0 |  |
|  |  |  |  |  |  |  |  |  |  |  |
| **Difference (B)-(A)** | SILVAMP-LAM specificity |  |  |  |  |  |  |  | -0.4 |  |
|  | LF-LAM specificity |  |  |  |  |  |  |  | -0.8 |  |
|  | Difference Δ Sp |  |  |  |  |  |  |  | 0.4 |  |
|  |  |  |  |  |  |  |  |  |  |  |
| **CRS** |  |  |  |  |  |  |  |  |  |  |
| **(A) Original Analysis** | |  |  |  |  |  |  |  |  |  |
| **CRS positive (Definite TB, Possible TB) / CRS negative (Not TB)** | | | | |  |  |  |  |  |  |
|  | **Test** | **N** | **TP** | **FP** | **FN** | **TN** | **Sn %** | **95% CI** | **Sp %** | **95% CI** |
| **All HIV+ CRS** | SILVAMP-LAM | 1595 | 570 | 47 | 264 | 714 | 65.8 | [55.9 - 74.6] | 93.4 | [89.3 - 96.2] |
|  | LF-LAM | 1595 | 323 | 25 | 511 | 736 | 31.4 | [19.1 - 43.7] | 97.3 | [95.1 - 98.9] |
|  | Δ Sn and Δ Sp |  |  |  |  |  | 34.4 |  | -3.8 |  |
|  |  |  |  |  |  |  |  |  |  |  |
| **(B) Sensitivity Analysis** | |  |  |  |  |  |  |  |  |  |
| **CRS positive (Definite TB, Possible TB, Unclassifiable) / CRS negative (Not TB)** | | | | | |  |  |  |  |  |
|  | **Test** | **N** | **TP** | **FP** | **FN** | **TN** | **Sn %** | **95% CI** | **Sp %** | **95% CI** |
| **All HIV+ CRS** | SILVAMP-LAM | 1795 | 592 | 47 | 442 | 714 | 53.6 | [37.9 - 69.2] | 93.4 | [77.2 - 96.5] |
|  | LF-LAM | 1795 | 341 | 25 | 693 | 736 | 26.7 | [17.2 - 36.8] | 97.6 | [90 - 99.4] |
|  | Δ Sn and Δ Sp |  |  |  |  |  | 26.9 |  | -4.2 |  |
|  |  |  |  |  |  |  |  |  |  |  |
| **Difference (B)-(A)** | SILVAMP-LAM sensitivity |  |  |  |  |  | -12.2 |  |  |  |
|  | LF-LAM sensitivity |  |  |  |  |  | -4.7 |  |  |  |
|  | Difference Δ Sn |  |  |  |  |  | -7.5 |  |  |  |
