# Supplementary material for: Diagnostic accuracy of a novel tuberculosis point-of-care urine lipoarabinomannan assay for people living with HIV: A meta-analysis of individual in- and outpatient data
Source: PLoS Med. 2020 May 1;17(5):e1003113. doi: 10.1371/journal.pmed.1003113 (PMC7194366; doi:10.1371/journal.pmed.1003113)
Supplement: S11 Table — (DOCX) [file pmed.1003113.s016.docx]

# S11 Table. Further Information on patients categorized as “not TB” with positive SILVAMP-LAM results

| **Cohort** | **Age (years)** | **Sex** | | **CD4 (cells/µL)** | | **WHO symptom screen** | | **TB History (time prior to enrollment)** | | **CRP (mg/l)** | | **Hb (g/dl)** | **Sputum *M.tb* Xpert and Culture** | | **Blood *M.tb* Culture** | | **Urine *M.tb* Xpert** | | **Clinical follow-up (8-12 weeks)** | | | **ART** | | | **Alternate diagnosis and clinical information** | | | **LF-LAM** | | |
| --- | --- | --- | --- | --- | --- | --- | --- | --- | --- | --- | --- | --- | --- | --- | --- | --- | --- | --- | --- | --- | --- | --- | --- | --- | --- | --- | --- | --- | --- | --- |
| 1A | 29 | Female | 21 | | Positive | | | yes  (~4 months) | | UK | | UK | Xpert negative Culture negative | | Negative | | Negative | | Alive improved | | yes | | | none | | | | | Negative | |
| 1A | 28 | Male | 12 | | Positive | | | no | | UK | | UK | Xpert negative Culture negative | | Negative | | Negative | | Alive improved | | no | | | none | | | | | Negative | |
| 1A | 46 | Male | 2 | | Positive | | | no | | UK | | UK | Xpert negative Culture negative | | Negative | | Negative | | Alive improved | | no | | | none | | | | | Negative | |
| 1B | 39 | Female | 127 | | Positive | | | no | | UK | | UK | Xpert negative Culture negative | | Negative | | Negative | | Alive improved | | yes | | | none | | | | | Negative | |
| 1B | 21 | Female | 609 | | Positive | | | no | | UK | | UK | Xpert negative Culture negative | | Negative | | Negative | | Alive improved | | yes | | | none | | | | | Positive | |
| 1B | 50 | Female | 386 | | Positive | | | no | | UK | | UK | Xpert negative Culture negative | | Negative | | Negative | | Alive improved | | yes | | | none | | | | | Positive | |
| 1B | 26 | Female | 212 | | Positive | | | yes | | UK | | UK | Xpert negative Culture negative | | Negative | | Negative | | Alive improved | | yes | | | none | | | | | Negative | |
| 1B | 29 | Female | 869 | | Positive | | | no | | UK | | UK | Xpert negative Culture negative | | Negative | | Negative | | Alive improved | | no | | | none | | | | | Negative | |
| 1B | 46 | Female | 1044 | | Positive | | | no | | UK | | UK | Xpert negative Culture negative | | Negative | | Negative | | Alive improved | | yes | | | none | | | | | Negative | |
| 2 | 39 | Female | 136 | | Positive | | | yes  (~1 year) | | 9.2 | | 9 | Could not provide sputum | | Negative | | Negative | | Alive | | yes | | | TB not suspected, acute illness. Patient presents with acute stroke (R-sided CVA) | | | | | Negative | |
| 2 | 50 | Male | 49 | | Positive | | | no | | 141 | | 7 | Xpert negative | | Negative | | Negative | | Alive | | naïve | | | Evidence of NTM on 2 sputum cultures. TB clinically suspected and presented with acute illness, CKD 2. Had Streptococcus pneumoniae in blood and Pneumonia-like picture | | | | | Negative | |
| **Cohort** | **Age (years)** | **Sex** | | **CD4 (cells/µL)** | | | **WHO symptom screen** | | **TB History (time prior to enrollment)** | | **CRP (mg/l)** | **Hb (g/dl)** | | **Sputum *M.tb* Xpert and Culture** | | **Blood *M.tb* Culture** | | **Urine *M.tb* Xpert** | | **Clinical follow-up (8-12 weeks)** | | | **ART** | | | **Alternate diagnosis and clinical information** | **LF-LAM** | | |  |
| 2 | 47 | Male | | 40 | | | Positive | | yes  (time unknown) | | 338 | 10.9 | | Xpert negative Culture negative | | Negative | | Negative | | Alive | | | interrupted | | | TB clinically suspected and presented with acute illness. Clinically felt to be CAP.  Diagnosis: Right lobar consolidation | Negative | | |  |
| 2 | 58 | Male | | 52 | | | Positive | | yes  (~1 year) | | 112 | 7.7 | | Xpert negative Culture negative | | Negative | | Negative | | Alive | | | interrupted | | | TB clinically suspected with chronic illness. Illness felt to be likely LRTI, although TB in differential diagnosis | Negative | | |  |
| 2 | 41 | Female | | 406 | | | Positive | | yes  (~2 years) | | 7 | 13.8 | | Could not provide sputum | | Negative | | Negative | | Alive | | | interrupted | | | Interrupted ART. TB not clinically suspected, subacute illness. CKD 3. Admission due to psychosis. | Negative | | |  |
| 2 | 35 | Male | | 252 | | | Positive | | yes  (~2 years) | | 2 | 14 | | could not provide sputum | | Negative | | Negative | | Alive | | | interrupted | | | Interrupted ART. TB not clinically suspected, acute illness. Likely neurocysticercosis. | Negative | | |  |
| 3 | 28 | Female | | 10 | | | Positive | | yes  (time unknown) | | 241 | 7.9 | | Xpert negative Culture negative | | not done | | Negative | | Deteriorated | | | yes | | | *E.coli* sepsis (blood and urine cult). Completed 5 days treatment and was discharged. During follow up, treated for CAP which did not require admission | Negative | | |  |
| 3 | 31 | Male | | 44 | | | Positive | | yes  (time unknown) | | 261 | 9.8 | | Xpert negative Culture negative | | Negative | | Negative | | Alive improved | | | UK | | | CAP | Negative | | |  |
| 4 | 34 | Female | | 321 | | | Positive | | yes | | UK | UK | | Xpert negative Culture negative | | not done | | not done | | Alive improved | | | yes | | | none | Negative | | |  |
| 4 | 38 | Male | | 7 | | | Positive | | no | | UK | UK | | Xpert negative Culture negative | | not done | | not done | | Alive improved | | | no | | | Cough improved with 4 weeks of sulfatrim treatment | Negative | | |  |
| 4 | 36 | Male | | 34 | | | Positive | | no | | UK | UK | | Xpert negative Culture negative | | not done | | not done | | Alive improved | | | no | | | After 1 week of unknown antibiotic treatment cough was improved | Negative | | |  |
| 4 | 33 | Male | | 52 | | | Positive | | no | | UK | UK | | Xpert negative Culture negative | | not done | | not done | | Alive improved | | | no | | | Cough did not improve after 1 week of azithromycin treatment, steroids yes | Negative | | |  |
| 5 | 30 | Male | | 25 | | | Positive | | no | | UK | 10.0 | | Xpert NA Culture negative | | not done | | not done | | Died (Day 98) | | | yes | | |  | Positive | | |  |
| 5 | 35 | Female | | 38 | | | Positive | | no | | UK | 11.1 | | Xpert NA Culture negative | | not done | | not done | | Died (Day 110) | | | yes | | |  | Negative | | |  |

| **Cohort** | **Age (years)** | | **Sex** | | | **CD4 (cells/µL)** | | | **WHO symptom screen** | **TB History (time prior to enrollment)** | **CRP (mg/l)** | **Hb (g/dl)** | **Sputum *M.tb* Xpert and Culture** | | | **Blood *M.tb* Culture** | | **Urine *M.tb* Xpert** | | **Clinical follow-up (8-12 weeks)** | | | **ART** | | **Alternate diagnosis and clinical information** | | **LF-LAM** | | |  |  |  |
| --- | --- | --- | --- | --- | --- | --- | --- | --- | --- | --- | --- | --- | --- | --- | --- | --- | --- | --- | --- | --- | --- | --- | --- | --- | --- | --- | --- | --- | --- | --- | --- | --- |
| 5 | 51 | | Male | | | 3 | | | Positive | no | UK | 8.0 | Xpert NA Culture negative | | | not done | | not done | | Alive | | | no | |  | | Positive | | |  |  |  |
| 5 | 43 | | Female | | | . | | | Positive | no | UK | 5.1 | Xpert NA Culture negative | | | not done | | not done | | Died (Day 91) | | | yes | |  | | Negative | | |  |  |  |
| 5 | 34 | | Female | 42 | | | Positive | | | No | UK | 8.1 | Xpert NA Culture negative | | | not done | | not done | | Alive | | yes | | |  | Negative | | | | |  |  |
| 5 | 28 | | Male | 65 | | | Positive | | | No | UK | 13.0 | Xpert NA Culture negative | | | not done | | not done | | Alive | | yes | | | *M. intracellulare-complex* | Negative | | | | |  |  |
| 5 | 35 | | Female | 8 | | | Positive | | | Yes | UK | 9.1 | Xpert NA Culture negative | | | not done | | not done | | Alive | | yes | | |  | Negative | | | | |  |  |
| 5 | 60 | | Female | 410 | | | Positive | | | No | UK | 11.1 | Xpert NA Culture negative | | | not done | | not done | | Alive | | no | | |  | Positive | | | | |  |  |
| 5 | 40 | | Male | 3 | | | Positive | | | No | UK | 11.0 | Xpert NA Culture negative | | | not done | | not done | | Alive | | yes | | |  | Negative | | | | |  |  |
| 5 | 47 | | Female | 397 | | | Positive | | | No | UK | 9.0 | Xpert NA Culture negative | | | not done | | not done | | Alive | | yes | | |  | Negative | | | | |  |  |
| 5 | 46 | | Female | 7 | | | Positive | | | No | UK | 8.1 | Xpert NA Culture negative | | | not done | | not done | | Alive | | yes | | |  | Negative | | | | |  |  |
| 5 | 26 | | Female | 4 | | | Positive | | | No | UK | 7.1 | Xpert NA Culture negative | | | not done | | not done | | Alive | | yes | | | Died on day 248 | Negative | | | | |  |  |
| 5 | 30 | | Male | 2 | | | Positive | | | No | UK | 10.1 | Xpert NA Culture negative | | | not done | | not done | | Alive | | yes | | | *M.avium* | Negative | | | | |  |  |
| 5 | 36 | | Female | 4 | | | Positive | | | No | UK | 12.1 | Xpert NA Culture negative | | | not done | | not done | | Alive | | yes | | |  | Negative | | | | |  |  |
| 5 | 36 | | Female | 6 | | | Positive | | | No | UK | 10.0 | Xpert NA Culture negative | | | not done | | not done | | Alive | | yes | | |  | Negative | | | | |  |  |
| **Cohort** | **Age (years)** | | **Sex** | **CD4 (cells/µL)** | | | | | **WHO symptom screen** | **TB History (time prior to enrollment)** | **CRP (mg/l)** | **Hb (g/dl)** | **Sputum *M.tb* Xpert and Culture** | | **Blood *M.tb* Culture** | | **Urine *M.tb* Xpert** | | **Clinical follow-up (8-12 weeks)** | | **ART** | | | | **Alternate diagnosis and clinical information** | | | **LF-LAM** | | | |  |
| 5 | 42 | | Male | 2 | | | | | Positive | No | UK | 13.0 | Xpert NA Culture negative | | not done | | not done | | LTFU | | yes | | | | Pneumocystis | | | Negative | | | |  |
| 5 | 47 | | Male | 16 | | | | | Positive | No | UK | 8.0 | Xpert NA Culture negative | | not done | | not done | | Alive | | yes | | | |  | | | Negative | | | |  |
| 5 | 38 | | Female | 8 | | | | | Positive | No | UK | 10.0 | Xpert NA Culture negative | | not done | | not done | | Alive | | yes | | | |  | | | Negative | | | |  |
| 5 | | 42 | Male | | 4 | | | Positive | | No | UK | 9.1 | | Xpert NA Culture negative | | not done | | not done | | Alive | | | | no |  | | | | Negative | | | |
| 5 | | 34 | Male | | 12 | | | Positive | | No | UK | 9.1 | | Xpert negative Culture negative | | not done | | not done | | Alive | | | | no |  | | | | Negative | | | |
| 5 | | 60 | Male | | 7 | | | Positive | | No | UK | 11.0 | | Xpert NA Culture negative | | not done | | not done | | Alive | | | | yes |  | | | | Negative | | | |
| 5 | | 30 | Female | | 67 | | | Positive | | No | UK | 6.0 | | Xpert negative Culture negative | | not done | | not done | | Alive | | | | yes |  | | | | Positive | | | |
| 5 | | 34 | Male | | 2 | | | Positive | | No | UK | 12.0 | | Xpert negative Culture negative | | not done | | not done | | Alive | | | | yes |  | | | | Negative | | | |
| 5 | | 43 | Male | | 75 | | | Positive | | Yes | UK | 4.1 | | Xpert negative Culture negative | | not done | | not done | | Alive | | | | yes | Started on TB treatment the Day 64 | | | | Negative | | | |
| 5 | | 32 | Female | | 71 | | | Positive | | No | UK | 7.0 | | Xpert negative Culture negative | | not done | | not done | | LTFU | | | | yes |  | | | | Negative | | | |
| 5 | | 44 | Male | | 48 | | | Positive | | No | UK | 11.1 | | Xpert negative Culture negative | | not done | | not done | | Alive | | | | yes | Kaposi sarcoma | | | | Negative | | | |

Abbreviations; UK–Unknown; CRP–C-reactive protein; CVA–Cerebrovascular accident; ART–antiretroviral therapy; NTM–Nontuberculous mycobacteria; CAP–community acquired pneumonia; CKD–chronic kidney disease; Hb–haemoglobin; LRTI–lower respiratory tract infection; TB–tuberculosis
