# Supplementary material for: Diagnostic accuracy of a novel tuberculosis point-of-care urine lipoarabinomannan assay for people living with HIV: A meta-analysis of individual in- and outpatient data
Source: PLoS Med. 2020 May 1;17(5):e1003113. doi: 10.1371/journal.pmed.1003113 (PMC7194366; doi:10.1371/journal.pmed.1003113)
Supplement: S12 Table — (DOCX) [file pmed.1003113.s017.docx]

#

# S12 Table. SILVAMP-LAM failure rates and errors for all samples tested

| **Normal runs, first attempt** | | **1801** | **100.00%** |
| --- | --- | --- | --- |
|  |  |  |  |
| **Failure on 1st try, no interpretation possible** | | **26** | **1.44%** |
|  | No control line, repeated | 12 | 0.50% |
|  | User error, repeated | 5 | 0.67% |
|  | Shadow in reading window, repeated | 1 | 0.06% |
|  | Shadow on reading window, not repeated by lab staff, excluded from analysis* | 3 | 0.16% |
|  | Liquid did not move, repeated | 2 | 0.11% |
|  | Button 3 failure, repeated | 1 | 0.06% |
|  | Liquid run back, repeated | 1 | 0.06% |
|  | No “Go Next” mark | 1 | 0.06% |
|  |  |  |  |
| **Repeat runs, second attempt** | | **23**§ | **100.00%** |
| **Failure on repeat, no interpretation possible** | | **3** | **13.00%** |
|  | No control line, not repeated, excluded from analysis | 2 | 8.70% |
|  | Liquid did not move, not repeated, excluded from analysis | 1 | 4.30% |

§ Second attempt for three samples was not possible because of insufficient sample volume

Conclusion: SILVAMP-LAM errors led to the exclusion of 6 patients from the analysis (3 because test repeat was not possible, and 3 because of test failure on repeat)

| **Total tests run** | | **1824** | **100.00%** |
| --- | --- | --- | --- |
|  | Normal runs | **1801** | 98.70% |
|  | Failure on 1st try, no interpretation possible | 26 | 1.4% |
|  | Repeats | 23§ | 1.3% |
|  | Failure on repeat, no interpretation possible | 3 | 0.16% |

§ Second attempt for three samples was not possible because of insufficient sample volume
