# Supplementary material for: Diagnostic accuracy of a novel tuberculosis point-of-care urine lipoarabinomannan assay for people living with HIV: A meta-analysis of individual in- and outpatient data
Source: PLoS Med. 2020 May 1;17(5):e1003113. doi: 10.1371/journal.pmed.1003113 (PMC7194366; doi:10.1371/journal.pmed.1003113)
Supplement: S13 Table — (DOCX) [file pmed.1003113.s018.docx]

# S13 Table. Agreement of two independent test readers for all samples tested

| **SILVAMP-LAM All PLHIV** | |  |  | **LF-LAM All PLHIV** | |  |
| --- | --- | --- | --- | --- | --- | --- |
|  |  |  |  |  |  |  |
| N=1594 | R2+ | R2- |  | N=1593 | R2+ | R2- |
| R1+ | 597 | 14 |  | R1+ | 329 | 18 |
| R1- | 24 | 959 |  | R1- | 23 | 1223 |
|  |  |  |  |  |  |  |
| p0 | 0.98 |  |  | p0 | 0.97 |  |
| kappa | 0.95 | [0.93-0.97] |  | kappa | 0.92 | [0.90-0.95] |
|  |  |  |  |  |  |  |
