# Supplementary material for: Diagnostic accuracy of a novel tuberculosis point-of-care urine lipoarabinomannan assay for people living with HIV: A meta-analysis of individual in- and outpatient data
Source: PLoS Med. 2020 May 1;17(5):e1003113. doi: 10.1371/journal.pmed.1003113 (PMC7194366; doi:10.1371/journal.pmed.1003113)
Supplement: S1 Translation — (DOCX) [file pmed.1003113.s019.docx]

**S1 Translation. Abstract in Japanese language**

**タイトル（Title）**

HIV感染結核患者の尿中リポアラビノマンナン抗原検出用新規簡易検査の診断精度評価：外来及び入院患者データのメタアナリシス

**要約（Abstract）**

背景

結核はHIV感染患者の死因として最大の感染症であるが、多くの患者が喀痰を喀出できず、定型的抗酸菌検査は高額でアクセスも容易でないため、未だにしばしば過小診断されている。新規迅速薄層免疫クロマトグラフィー法である富士フイルムSILVAMP TB LAM (SILVAMP-LAM)は、尿中の結核菌リポアラビノマンナン(LAM)を検出し、既に使用されているLAMキット（LF-LAM）よりもHIV合併結核患者の診断に関して大幅に感度が向上している。そこで我々は、成人のHIV感染者におけるSILVAMP-LAMの診断精度に関する個別患者データメタアナリシスを、既に報告されているものと未報告のものを含めて提示する。

方法と結果（Methods and Findings）

2012年〜2017年の間に南アフリカ（3研究）、ベトナム（1研究）及びガーナ（1研究）で実施された5つの前向きコホート研究において、成人のHIV感染者（18歳以上）を対象とした。組み入れ基準に合致した1,595名のHIV感染者のうち、大半（61%）は入院患者であり、平均年齢は39歳［四分位範囲/IQR 30–43］であった。43%はCD4陽性細胞数が100/µl以下であり、35%は抗レトロウイルス療法を受けていた。殆どの患者（94%）は組み入れ時にWHOの結核症候スクリーニングで陽性であり、45%は喀痰、尿あるいは血液を用いた抗酸菌培養検査あるいはXpert MTB/RIF検査で細菌学的に結核の診断がついていた。既に報告済みの入院患者データと未発表の外来患者データを組み合わせて解析に用いた。これらの患者から採取され保存されていた尿検体を使用し、二重盲検法でSILVAMP-LAMとLF-LAMを比較した。

細菌学的なデータを感度の参照基準として適用した場合、総合的感度はSILVAMP-LAMで70.7% (95%CI: 59.0–80.8)、LF-LAMで34.9% (19.5–50.9)であった。総合診断結果（臨床診断と細菌学的診断を併せた患者総数）を参照値とした場合は、SILVAMP-LAMの感度は65.8% (55.9–74.6)で、LF-LAMでは31.4% (19.1–43.7)であった。CD4陽性細胞数100/µl以下の患者の場合、LF-LAM の感度が56.0% (43.9–64.9)であったのに対して、SILVAMP-LAMの感度は87.1% (79.3–93.6) であった。CD4陽性細胞数100–200/µlの患者の場合は、LF-LAM の感度が25.3% (15.8–34.9)であったのに対して、SILVAMP-LAMの感度は62.7% (52.4–71.9)であった。CD4陽性細胞数200/µl超の患者の場合はLF-LAM の感度が10.9% (5.2–18.4)であったのに対して、SILVAMP-LAMの感度は43.9% (34.3–53.9)であった。細菌学的診断を参照基準とした場合、SILVAMP-LAMの特異度は90.9% (87.2–93.7)であり、LF-LAMは95.3% (92.2–97.7)であった。

この研究の限界は、新鮮な尿ではなく保存検体を用いていることと、ポイントオブケア検査と言うには、研究施設内で技術的に優れた検査者が実施しているという点である。

結論（Conclusion）

この研究から、我々はHIV合併結核患者において、SILVAMP-LAMはLF-LAMよりも遙かに多くの患者を同定できることを示した。CD4陽性細胞数100/µl以下の患者でSILVAMP-LAMの感度は最も高かった。ポイントオブケア検査としての精度を明らかにするため、さらなる研究が必要である。
