# Supplementary material for: Diagnostic accuracy of a novel tuberculosis point-of-care urine lipoarabinomannan assay for people living with HIV: A meta-analysis of individual in- and outpatient data
Source: PLoS Med. 2020 May 1;17(5):e1003113. doi: 10.1371/journal.pmed.1003113 (PMC7194366; doi:10.1371/journal.pmed.1003113)
Supplement: Attachment — Submitted filename: broger.pdf [file pmed.1003113.s020.pdf]

This paper presents a diagnostic meta–analysis of two diagnostic tests for TB in people living with HIV,

I was asked for a statistical report and I interpret that to include all aspects of the design and conduct of the study.

Points of detail

Page 1 Perhaps mention TB in the title?

Page 7 I do not understand the sentence which starts 'A random selection method'. I would remark that using mechanical devices like a die to generate random numbers is problematic if that is indeed what was done. Search on–line for casino die specification for details of the lengths you need to go to to make a fair die.

Page 12 I did not grasp why Possible TB is considered differently against the two reference standards. If this is immediately obvious to TB specialists then fair enough but otherwise a sentence of explanation would help.

Page 12 If the authors used a contributed package in R I think a proper citation should be made. It is the only recognition authors get.

Page 12 Strictly speaking random effects does not 'account' for heterogeneity, it just estimates a different model. See Rice et al. (2018) for discussion in the context of the univariate meta–analysis.

Page 14, Table 1 The shading does not really add any extra scientific meaning and just makes it harder to read.

Page 16 There seem to be rather different exclusion rates in the cohorts. This perhaps needs some discussion especially the unclassifiables.

Page 18 Is the influence of CD4 count the outcome of a formal meta–regression or something else?

If the authors are taking a Bayesian approach I would have expected to see credible intervals rather than confidence intervals.

Points of more substance

Heterogeneity

Looking at Figure S1 we see that the sensitivities vary considerably between the cohorts. A $\chi^2_5 = 36.7$ for FujiLAM using MRS suggests this would be thought statistically significant beyond most conventional levels. I have not checked for AlereLAM but given the lamentable performance for it in Cohort 1B I imagine the same might apply. Why is the performance of the test so variable between cohorts and does this question the wisdom of presenting aggregates of performance?

What is the nature of the test differences?

The authors are comparing the two tests in terms of the standard techniques of sensitivity and specificity. This does not let us see how the difference arises. For instance are the 307 true positives detected by AlereLAM a proper subset of the 541 detected by FujiLAM and so on?

Relationship between Tango and meta–analysis

I do not understand how the authors used the results from their meta–analysis to provide input for Tango's method which relies, if I understand it correctly, on having a 2×2 table which is not what the output from a diagnostic meta–analysis provides.

Summary

Some clarifications needed about the analysis.

Michael Dewey

References

K Rice, J P T Higgins, and T Lumley. A re–evaluation of fixed effect(s) meta–analysis. *Journal of the Royal Statistical Society*, 181:205–227, 2018.
